# Response to Interleukin-1 Inhibitors in 140 Italian Patients with Adult-Onset Still’s Disease: A Multicentre Retrospective Observational Study

**DOI:** 10.3389/fphar.2017.00369

**Published:** 2017-06-13

**Authors:** Serena Colafrancesco, Roberta Priori, Guido Valesini, Lorenza Argolini, Elena Baldissera, Elena Bartoloni, Daniele Cammelli, Giovanni Canestrari, Luca Cantarini, Elena Cavallaro, Giulio Cavalli, Lucia Cerrito, Paola Cipriani, Lorenzo Dagna, Ginevra De Marchi, Salvatore De Vita, Giacomo Emmi, Gianfranco Ferraccioli, Micol Frassi, Mauro Galeazzi, Roberto Gerli, Roberto Giacomelli, Elisa Gremese, Florenzo Iannone, Giovanni Lapadula, Giuseppe Lopalco, Raffaele Manna, Alessandro Mathieu, Carlomaurizio Montecucco, Marta Mosca, Ilaria Piazza, Matteo Piga, Irene Pontikaki, Micol Romano, Silvia Rossi, Maurizio Rossini, Piero Ruscitti, Elena Silvestri, Chiara Stagnaro, Rosaria Talarico, Angela Tincani, Ombretta Viapiana, Gianfranco Vitiello, Francesca Fabris, Sara Bindoli, Leonardo Punzi, Paola Galozzi, Paolo Sfriso

**Affiliations:** ^1^Rheumatology Unit, Department of Internal Medicine and Medical Specialties, Sapienza University of RomeRome, Italy; ^2^Division of Rheumatology, ASST Gaetano PiniMilan, Italy; ^3^Unit of Immunology, Rheumatology, Allergy and Rare Diseases (UnIRAR), IRCCS San Raffaele Scientific InstituteMilan, Italy; ^4^Rheumatology Unit, Department of Medicine, University of PerugiaPerugia, Italy; ^5^Department of Experimental and Clinical Medicine, University of FlorenceFlorence, Italy; ^6^Rheumatology Section/Immunoallergology Unit, AOU CareggiFlorence, Italy; ^7^Institute of Rheumatology and Affine Sciences, Division of Rheumatology, Catholic University of the Sacred HeartRome, Italy; ^8^Research Center of Systemic Autoinflammatory Diseases and Behçet’s Disease Clinic Surgery and Neurosciences, Department of Medical Sciences, Surgery and Neurosciences, University of SienaSiena, Italy; ^9^Department of Medical and Biological Sciences, Rheumatology Clinic, University of UdineUdine, Italy; ^10^Department of Internal Medicine, Vita-Salute San Raffaele UniversityMilan, Italy; ^11^Periodic Fever Research Center, Institute of Internal Medicine, Catholic University of the Sacred Heart, Fondazione Policlinico A. GemelliRome, Italy; ^12^Department of Biotechnological and Applied Clinical Science, Division of Rheumatology, University of L’AquilaL’Aquila, Italy; ^13^Rheumatology and Clinical Immunology, Spedali Civili and Department of Clinical and Experimental Sciences, University of BresciaBrescia, Italy; ^14^Rheumatology Unit, Interdisciplinary Department of Medicine, University of BariBari, Italy; ^15^Rheumatology Unit, Department of Medical Sciences, University and AOU of CagliariCagliari, Italy; ^16^Department of Rheumatology, IRCCS Policlinico San Matteo Foundation, University of PaviaPavia, Italy; ^17^Rheumatology Unit, Department of Clinical and Experimental Medicine, University of PisaPisa, Italy; ^18^Rheumatology Unit, Department of Medicine, University of VeronaVerona, Italy; ^19^Department of Medicine DIMED, Rheumatology Unit, University of PaduaPadua, Italy

**Keywords:** Adult-onset Still’s disease, treatment, interleukin (IL)-1, anakinra, canakinumab

## Abstract

**Background:** Interleukin (IL)-1 plays a crucial role in the pathogenesis of Adult onset Still’s disease (AOSD).

**Objectives:** To evaluate the efficacy and safety of anakinra (ANA) and canakinumab (CAN) in a large group of AOSD patients.

**Methods:** Data on clinical, serological features, and concomitant treatments were retrospectively collected at baseline and after 3, 6, and 12 months from AOSD patients (Yamaguchi criteria) referred by 18 Italian centers. Pouchot’s score was used to evaluate disease severity.

**Results:** One hundred forty patients were treated with ANA; 4 were subsequently switched to CAN after ANA failure. The systemic pattern of AOSD was identified in 104 (74.2%) of the ANA-treated and in 3 (75%) of the CAN-treated groups; the chronic-articular type of AOSD was identified in 48 (25.8%) of the ANA-treated and in 1 (25%) of the CAN-treated groups. Methotrexate (MTX) was the most frequent disease modifying anti-rheumatic drug (DMARD) used before beginning ANA or CAN [91/140 (75.8%), 2/4 (50%), respectively]. As a second-line biologic DMARD therapy in 29/140 (20.7%) of the patients, ANA was found effective in improving all clinical and serological manifestations (*p* < 0.0001), and Pouchot’s score was found to be significantly reduced at all time points (*p* < 0.0001). No differences in treatment response were identified in the ANA-group when the patients were stratified according to age, sex, disease pattern or mono/combination therapy profile. ANA primary and secondary inefficacy at the 12-month time point was 15/140 (10.7%) and 11/140 (7.8%), respectively. Adverse events (AEs) [mainly represented by in situ (28/47, 59.5%) or diffuse (12/47, 25.5%) skin reactions and infections (7/47, 14.8%)] were the main causes for discontinuation. Pouchot’s score and clinical and serological features were significantly ameliorated at all time points (*p* < 0.0001) in the CAN-group, and no AEs were registered during CAN therapy. Treatment was suspended for loss of efficacy only in one case (1/4, 25%).

**Conclusion:** This is the largest retrospective observational study evaluating the efficacy and safety of IL-1 inhibitors in AOSD patients. A good response was noted at 3 months after therapy onset in both the ANA- and CAN-groups. Skin reaction may nevertheless represent a non-negligible AE during ANA treatment.

## Introduction

Adult-onset Still’s disease (AOSD) is a rare multisystemic inflammatory disorder predominantly affecting young adults, with an estimated annual incidence of 0.16–0.4 per 100,000 persons globally (Jamilloux et al., 2015; Sfriso et al., 2016). Various genetic, infectious and environmental factors seem to interact triggering a systemic autoinflammatory response in predisposed individuals. A dysregulation of cytokine-mediated pathways, in particular those linked to interleukin (IL)-1, IL-6, IL-18, tumor necrosis factor-α (TNF-α), and interferon-γ (IFN-γ) (Fujii et al., 2001; Giampietro and Fautrel, 2012) has been hypothesized.

Adult-onset Still’s disease is clinically characterized by daily high spiking fever, evanescent maculopapular skin rash, arthritis, musculoskeletal symptoms, sore throat and hepatosplenomegaly. Cardiopulmonary manifestations and significant liver dysfunctions are only rarely present. Central nervous system (CNS) and renal involvement has, likewise, been described only in very few case reports (Gerfaud-Valentin et al., 2014a). Typical laboratory findings include marked leukocytosis with neutrophilia, hyperferritinemia, high liver enzymes, and elevated acute-phase reactants such as erythrocyte sedimentation rate (ESR) and C-reactive protein (CRP).

The clinical presentation of AOSD can be distinguished into two phenotypes: a highly symptomatic, feverish, systemic pattern and a chronic articular profile showing features of polyarthritis. AOSD treatment, which essentially remains empirical, is based on small retrospective case series studies (Jamilloux et al., 2015). Non-steroidal anti-inflammatory drugs (NSAIDs) and glucocorticoids are generally used as a first-line treatment, in particular for musculoskeletal manifestations and fever. Disease-modifying anti-rheumatic drugs (DMARDS), such as methotrexate, azathioprine, and leflunomide, are often used in the attempt to reduce the quantity of corticosteroids being administered. Intravenous immunoglobulin, anti-TNF-α drugs, such as etanercept, infliximab and adalimumab, as well as anti-IL-6 agents, i.e., tocilizumab, also appear to adequately control the disease in non-responders to conventional therapy (Cefle, 2005; Fautrel et al., 2005; Kim et al., 2012). These treatments may nevertheless show variable efficacy and/or may be linked to potentially severe side effects.

Increasing evidence regarding the global efficacy of IL-1 inhibitors (IL-1-INH), such as Anakinra (ANA; IL-1 receptor antagonist) and Canakinumab (CAN; monoclonal anti-IL-1β antibody) has been collected from refractory systemic and articular AOSD patients (Kontzias and Efthimiou, 2012; Ortiz-Sanjuán et al., 2015). ANA is a recombinant, non-glycosylated form of human IL-1 receptor antagonist; since it has a very short half-life, daily subcutaneous administrations are necessary. CAN, which is a human anti-IL-1β monoclonal antibody, has a longer half-life.

In August 2016, the European Commission extended the license approval of CAN to treat active Still’s disease including AOSD and Systemic Juvenile Idiopathic Arthritis (SJIA). The decision reached in the light of evidence supporting the concept of a Still’s disease continuum including both the juvenile and adult onset forms (Nirmala et al., 2015) and as the scientific comunity awaits the results of an ongoing trial (NCT02204293).

The current study aimed to examine the use of IL-1-INH in a large number of Italian patients with AOSD, found to be refractory to other therapies. A nationwide cross-sectional observational study promoted by the Italian Group of Study on Autoinflammatory Diseases and endorsed by the Italian Society of Rheumatology (SIR) was thus conducted to gather information about their efficacy and their effect on clinical features and inflammatory markers during treatment.

## Materials and Methods

### Patients and Data Collection

Demographic, clinical, and therapeutic data were retrospectively collected from AOSD patients attending 18 Italian University-Hospital centers. The patients were considered eligible if they were adults with AOSD diagnosed in accordance with Yamaguchi’s criteria (Yamaguchi et al., 1992) (**Table 1**) and being treated with IL-1-INH after failure of therapy based on NSAIDs and immunosuppressive drugs, such as steroids and DMARDs, and in some cases other biologic agents.

**Table 1:** Yamaguchi criteria (Yamaguchi et al., 1992).

Major criteria	Minor criteria
(1) Fever ≥39°C (≥1 week)	(1) Sore throat
(2) Arthralgia (≥2 weeks)	(2) Lymphadenopathy and/or splenomegaly
(3) Typical rash	(3) Liver dysfunction
(4) Leukocytosis (≥10 000/mm^3^) with ≥ 80% of granulocytes	(4) Negative RF and ANA


*Diagnosis requires ≥5 criteria including at least 2 or more major criteria. Infections, malignancies, and other rheumatic diseases must be excluded. RF, rheumatoid factor; ANA, antinuclear antibody.*

Attending physicians provided retrospective anonymous information from medical records, which were entered into a database, regarding the patients’ clinical and laboratory data, the therapies prescribed to manage AOSD including all those prior to IL-1-INH, response to ANA or CAN, adverse events (AEs), and the effect on clinical symptoms 3, 6, or 12 months after IL-1-INH therapy was begun.

The study was carried out in accordance with Good Clinical Practice, the Declaration of Helsinki and the recommendations of the local Ethical Committee rules of all participating centers.

### Definition of Clinical and Laboratory Criteria

In accordance with the established definition, the disease was considered the systemic form if the patient primarily showed marked increase in inflammatory markers, hyperferritinemia, and multi-organ involvement. The disease was considered the chronic form if involvement was prevalently polyarticular and the patient presented low levels of inflammatory markers and erosive damage. Disease severity was determined using a modified Pouchot’s score (range 0–12) (Rau et al., 2010), which considers 12 disease-related manifestations [fever, evanescent rash, pleuritis, pneumonia, pericarditis, hepatomegaly, serum ferritin levels (>3000 mg/L), lymphadenopathy, white blood cells count (>15,000/mm), sore throat, myalgias, and arthritis]. Fever was defined by temperatures ≥39°C; cutaneous rash was considered positive if the patients presented an evanescent salmon-pink, macular, or maculopapular rash predominantly on the trunk and limbs. Pleuritis was defined as pleural effusion linked with pleuritic pain; pericarditis was defined by chest pain, pericardial friction rub, and effusion documented by echocardiogram. Pneumonia was diagnosed in presence of pulmonary consolidations documented by X-rays or chest computed tomography (CT) scan. Lymphoadenopathy was confirmed by ultrasound and/or CT scan in at least two different sites. Diagnosis of hepatomegaly was confirmed by ultrasound and/or CT and/or nuclear magnetic resonance spectroscopy (NMR) scan findings. Leukocytosis was defined as a white blood cell count ≥15,000/mm^3^; hyperferritinemia was defined as serum ferritin ≥3000 ng/mL. ESR and CRP levels were considered elevated when values fell beyond the laboratory reference limit.

Response to anti IL-1 treatment was scored as complete, partial, or failure. Response was considered complete or provoking remission when signs of active disease were absent and inflammatory markers were normalized. It was considered partial when complete response was not achieved although there were clear signs of clinical improvement according to the attending physician.

### Statistical Analysis

D’Agostino–Pearson’s test for normality was used. The normally distributed variables were described by the mean ± standard deviation (SD), and the non-normally distributed variables using the median and range. Wilcoxon’s matched-pairs test and paired *t*-tests were performed. Pearson’s and Spearman’s tests were carried out to analyze the correlations where appropriate. Univariate analysis of nominal variables was carried out using the chi-square (χ^2^) test or Fisher’s exact-test where appropriate. The *P*-values of two-tailed tests were calculated; *p*-values less than or equal to 0.05 were considered significant.

The statistical calculations were performed using the Statistical Package for Social Sciences 13.0 (SPSS, Chicago, IL, United States).

## Results

The data from 140 AOSD patients (93 females and 47 males; mean age at disease onset = 35.4 ± 17 years, mean age at diagnosis = 37.4 ± 16.1 years) were evaluated. All the patients were treated with ANA; four were later switched to CAN after ANA failed. The mean disease duration before starting treatment with ANA was 50.33 ± 81.67 months. Most of the patients presented a systemic disease pattern (104/140, 74.2%) and the rest presented a chronic articular one (36/140, 25.8%).

### Anakinra Treatment

#### Previous or Concomitant Therapies

**FIGURE 1:**
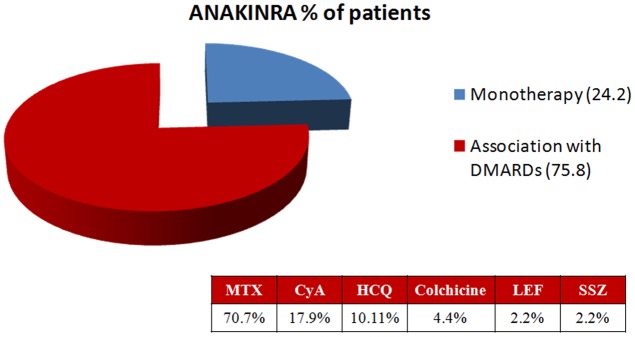
Concomitant therapies during ANA treatment. MTX, methotrexate; CyA, cyclosporine A; HCQ, hydroxychloroquine; LEF, leflunomide; SSZ, sulfasalazine.

Most of the patients were treated with NSAIDs, glucocorticoids and/or DMARDs before starting ANA (**Table 2**). In the majority of cases, steroids represented the first-line therapy (mean initial dosage of prednisone (PDN) equivalent of 77.6 ± 186.3 mg daily). DMARDs were employed in 120/140 (85.7%). Methotrexate (MTX) was used in 91/120 (75.8%) and cyclosporine A (CyA) in 50/120 cases (41.6%). ANA was adopted as a second-line biological therapy (bDMARD) in 29/140 patients (20.7%); it was the first-line biological treatment in 111/140 patients (79.3%). As far as bDMARDs are concerned, anti-TNF-α therapies represented the prevalent strategy and etanercept (ETN) and infliximab (IFX) were the most frequently used drugs (79.3 and 44.8%, respectively). In the majority of cases (106/140, 75.8%), ANA was prescribed in combination with other DMARDs; in 24.2% (34/140) it was used as a monotherapy (**Figure 1**). MTX represented the first choice DMARD used in association with ANA (**Figure 1**); in some cases (13/140, 9.2%), a combination of more than one DMARD was utilized.

**Table 2:** Previous therapies prescribed to the Adult onset Still’s disease (AOSD) patients before they began Anakinra (ANA) therapy.

Therapies	% of patients (*n* = 140)
**Non-steroidal Anti-Inflammatory Drugs (NSAIDs)**	69.2
**Steroids**	97.8
**Disease-Modifying Anti-Rheumatic Drugs (DMARDs)**	85.7
*Methotrexate (MTX)*	75.8
*Cyclosporine A (CyA)*	41.6
*Hydroxychloroquine (HCQ)*	22.1
*Colchicine*	9.2
*Azathioprine (AZA)*	6.4
*Salazopyrine (SSZ)*	5.7
*Leflunomide (LEF)*	3.5
**Biological therapies (bDMARDs)**	20.7
*Etanercept (ETN)*	79.3
*Infliximab (IFX)*	44.8
*Adalimumab (ADA)*	20.6
*Tocilizumab (TOCI)*	6.8
*Abatacept (ABA)*	6.8
*Golimumab (GOL)*	6.8
*Rituximab (RTX)*	6.8
*Certulizumab (CTZ)*	6.8


#### ANA Dosage

At baseline, 100 mg per day ANA was administered to 127/140 patients (90.7%); higher or lower doses were prescribed to 13/140 cases (9.3%). Four patients presented with a very aggressive systemic form of the disease; 3 (23.1%) of these were originally treated with 200 mg/day ANA and 1 (7.6%) was treated with 150 mg/day. In view of intolerability issues, nine patients were prescribed lower, non-conventional dosages, i.e., 50 mg per day (one case) or 100 mg every other day (eight cases).

Dosage was adjusted for 33/140 patients (23.5%) over the course of treatment. In 29/33 cases showing marked improvement, the dosage was reduced from 100 mg per day to 100 mg every other day. In 3/33 cases therapy was upgraded to the standard 100 mg/day dose. In a single case, therapy was increased from 100 mg to 200 mg per day in view of an incomplete clinical response.

#### Duration of Therapy, Discontinuation, and AEs

After 12 months of treatment, 97/140 patients (69.2%) were still receiving ANA. Overall, the mean duration of therapy was 35.7 ± 36.1 months. At the time, we analyzed the data, 69 out of 140 patients (49.3%) were still being treated with ANA, and 71 (50.7%) had discontinued therapy (**Table 3**).

**Table 3:** The number of patients receiving ANA during the study period.

	Baseline	3 months	6 months	12 months	Time of this study
N° of patients (%)	140 (100%)	118 (84.2%)	109 (77.8%)	97 (69.2%)	69 (49.2%)


**FIGURE 2:**
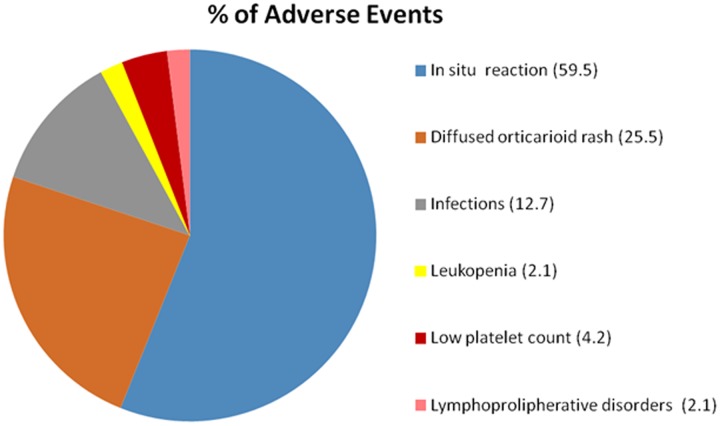
Adverse events (AEs) linked to ANA.

The main reason for treatment discontinuation was linked to the development of AEs (24/71 patients, 33.8%) followed by remission (sustained disappearance of all clinical and serological manifestations) in 20/71 cases (28.1%) (**Figure 2**). Discontinuation for primary inefficacy was documented in 16/71 subjects (22.5%). ANA was discontinued in 11/71 cases (15.4%) due to loss of efficacy during the follow-up.

AEs occurred in 47/140 patients (33.5%). The principal AE reported was the appearance of *in situ* (28/47 cases, 59.5%) or diffuse (12/47 cases, 25.5%) local reactions, usually in the form of cutaneous urticarial lesions. Most of the patients abandoning therapy because of AEs (18/24, 75%) did so because of severe skin reactions. Infectious events (7/47 patients, 12.7%) were quite rare: they consisted in three cases of pneumonia, three cases of urinary tract infections and one case of recurrent dental abscesses; infectious events caused withdrawal in two out of seven cases.

#### The Clinical Efficacy of ANA

**FIGURE 3:**
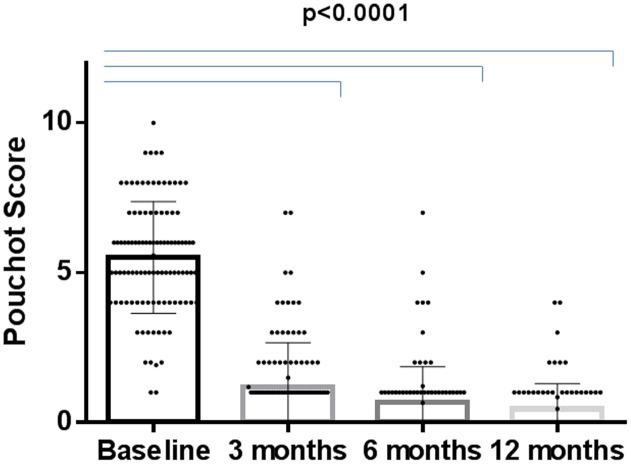
Changes in Pouchot’s score during the 12-months study period in the ANA-treated patients.

Anakinra proved to be effective in reducing all AOSD-linked clinical and serological manifestations. Primary and secondary inefficacy after 12 months was reported in 15/140 (10.7%) and 11/140 (7.8%) patients, respectively. Pouchot’s score, which was calculated at baseline and then at 3, 6, and 12 months, demonstrated a significant improvement; there was a drop in the mean score already at 3 months (5.5 ± 1.9, range 2–10, at baseline versus 1.1 ± 1.4, range 0–7, after 3 months; *p* < 0.0001) (**Figure 3**).

An analysis of the prevalence of the disease’s main clinical symptoms [fever, rash, pneumonia, pericarditis, pleuritis, sorethroat, lymphadenopathy, hepatomegaly, myalgia, arthritis, macrophage activation syndrome (MAS)] and laboratory features (increased liver enzymes, hyperferritinemia, leucocytosis) at 3, 6, and 12 months uncovered a significant reduction in all of these (*p* < 0.0001) beginning as early as 3 months into the follow-up (**Table 4**). There were 98/140 (70%) patients who were experiencing arthritis symptoms at baseline with a mean Disease Activity Score 28 (DAS28) score of 4.7 ± 1.2 (range 1.4–7.29) which fell significantly 3 months into therapy to 2.4 ± 1.08 (range 0.96–6.01) (*p* < 0.0001). After 12 months, the mean DAS28 score fell even further reaching 1.7 ± 0.9 (0.49–6.8).

**Table 4:** Clinical and laboratory features at baseline and during the 12-months study period in the ANA-treated patients.

Clinical and Laboratory	Baseline (*n* = 140) (%)	3 months (*n* = 118) (%)	6 months (*n* = 109) (%)	12 months (*n* = 97) (%)
Fever	96.4	12.7	10	1
Rash	73.5	9.3	4.2	3
Pleuritis	14.2	1.6	3.6	2
Pneumonia	7.1	0	0	0
Pericarditis	17.8	0.8	0	0
Lymphadenopathy	51.4	12.7	4.5	3
Hepatomegaly or increased liver enzymes	47.1	9.3	5.5	5
Hyperferritinaemia	67.8	10.1	2.7	2
Leucocytosis	70	8.4	3.6	2
Sore throat	54.2	5	2.7	3
Myalgia	75	33	18.3	13.4
Arthritis	69.2	33	15.5	14.4
Macrophage Activation Syndrome (MAS)	8.5	0.8	0.9	1


No differences in the clinical or serological response to treatment were identified when the patients were stratified according to age, sex or disease pattern (systemic or chronic articular). Nor were differences observed when the patients receiving ANA monotherapy were compared to those receiving ANA combined with DMARDs or when the patients previously treated with other biological drugs were compared with those naïve to biological therapy.

Twelve (8.5%) patients showed signs of MAS before they were prescribed ANA treatment. After therapy was begun, five cases of MAS presented: one after 3 months (the patient recovered without discontinuing ANA), one after 6 months (the patient fully recovered without discontinuing therapy), 2 after 12 months (one patient recovered and continued ANA therapy; the other died). Another case of fatal MAS occurred after 16 months of therapy and the patient underwent disseminated intravascular coagulation (DIC); then the patient was successfully reanimated after cardiac arrest. However, a new episode of MAS led to the death of the patient who was still receiving ANA treatment 30 months later.

In one case a patient who was one of the 20 (14.2%) who showed complete disease remission needed to begin ANA therapy again. The other 19 cases are still in remission (mean follow-up 56.8 ± 54 months): 9 (47.3%) are receiving only DMARDs therapy, while 10 (52.7%) are completely drug-free.

#### The Laboratory Efficacy of ANA

**FIGURE 4:**
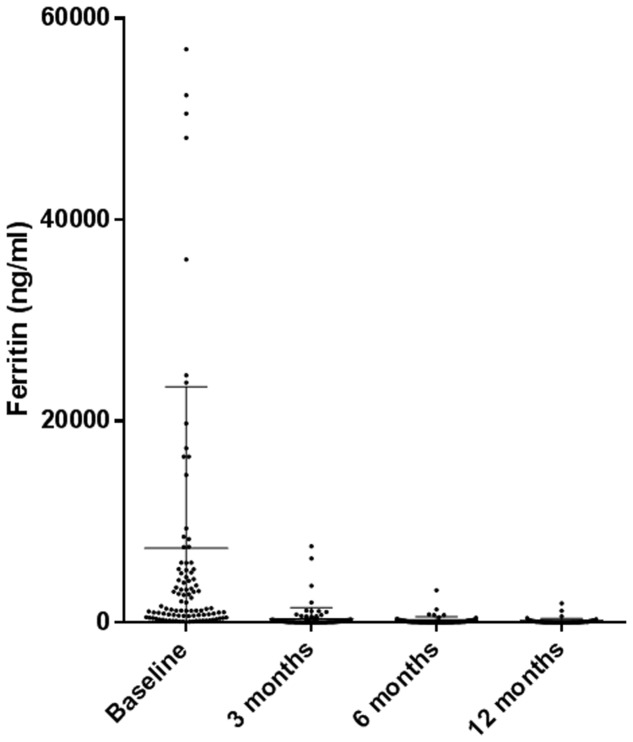
Modifications in ferritin levels during the 12-months study period in the ANA-treated patients.

Laboratory parameters were significantly modified by therapy. Ferritin serum levels were lower after 3 months of ANA with respect to baseline data [397.5 ± 1072.68 ng/ml (range 15–7581 ng/ml) versus 5965.97 ± 14677.48 ng/ml (range 43–105000 ng/ml); *p* < 0.0001] and other inflammatory markers including ESR and CRP showed improved levels (**Figure 4** and **Table 5**). Almost 33% of the patients experienced liver involvement which was confirmed by higher liver enzyme levels. Improvement was noted already after 3 months of therapy (4.2% patients, *p* < 0.0001).

**Table 5:** Laboratory features at baseline and during the 12-months study period in the ANA-treated patients.

Laboratory		Baseline (*n* = 140) (%)	3 months (*n* = 118) (%)	6 months (*n* = 109) (%)	12 months (*n* = 97) (%)
Ferritin					
>200 ng/ml		67.8	21.6	8.4	7.3
>1000 ng/ml		47.1	9.2	2.4	2.9
>3000 ng/ml		28.5	3	1.2	0
>10000 ng/ml		10	0	0	0
Eritrocyte sedimentation rate (ESR)		85	32.1	9.1	8.2
C-reactive protein (CRP)		90	30.5	12.8	8.2
Augmented liver enzymes		32.8	5	6.4	3


#### The Impact of ANA on Concomitant Therapies during the Follow-Up

Three months after beginning ANA therapy, the prevalence of patients also receiving steroids was not significantly different (97.8% patients at baseline versus 86.4% at the end of the third month, *p* > 0.05). The mean dosage was significantly lower (77.6 ± 186.3 mg of Prednisone (PDN) at baseline versus 8.8 ± 11.2 mg of PDN at the end of the third month; *p* < 0.0001). After 12 months, the percentage of patients receiving steroid therapy, which had fallen to 55.6%, was significantly different (*p* < 0.001) (**Table 6**). The percentage of patients receiving DMARD therapy had significantly fallen at the end of 12 months (85.7% patients at baseline versus 59.7% at the end of the 12th month, *p* < 0.001) and it fell even further (to 50.7%) at study completion (**Tables 6**, **7**).

**Table 6:** Concomitant therapy change during the 12-months study period in the ANA-treated patients.

Therapy	Baseline (*n* = 140)	3 months (*n* = 118)	6 months (*n* = 109)	12 months (*n* = 97)
Steroids	97.8%	86.4%	68.8%	55.6%
	77.6 ± 186.3 mg	8.8 ± 11.2 mg	5.2 ± 6.9 mg	3.4 ± 4.8 mg
DMARDs	85.7%	66.1%	59.6%	59.7%


**Table 7:** Anakinra and canakinumab (CAN) therapy status after 12 months of follow-up.

	ANA	CAN
Last Follow-up	32.2 ± 41.5 months	65.75 ± 76.34 months
Ongoing Therapy	49.2% (69/140) patients	50% (1/4) patients
Mean therapy duration	35.7 ± 36.1 months	22.1 ± 16.5 months
Ongoing steroids	31.8% (22/69) patients	100% (3/3) patients
Ongoing DMARDs	50.7% (35/69) patients	33.3% (1/3) patients


### Canakinumab Treatment

Four patients in whom ANA proved inefficacious were switched to CAN. The mean age of these patients at onset was 34.2 ± 15.4 years; the mean age at diagnosis was 34.7 ± 13.3 years. The mean duration of disease before starting CAN treatment was 58.33 ± 48.4 months. Three of these presented a systemic disease pattern and one a chronic articular profile.

#### Previous or Concomitant Therapies

Two patients were receiving CAN in association with other DMARDs, the other 2 were receiving monotherapy. The latter were treated previously with other DMARDs including MTX, hydroxychloroquine (HCQ) and CyA. Before starting ANA treatment, three patients were unsuccessfully treated with other bDMARDs: one was prescribed IFX, ETN, adalimumab, and tocilizumab; another tocilizumab; the third adalimumab.

#### CAN Dosage, Therapy Duration, Reasons for Discontinuing and AEs

Canakinumab was administered at the standard dose of 150 mg every 8 weeks without dose adjustment neither at the beginning nor during the follow-up period. The mean duration of therapy was 22.1 ± 16.5 months. Treatment is still ongoing in two patients. In the other two cases, it was discontinued; in one case, it was discontinued after 9 months because of loss of efficacy, in the other it was discontinued after 45 months because of remission. No AEs were registered in the CAN-treated patients.

#### CAN Clinical Efficacy

**FIGURE 5:**
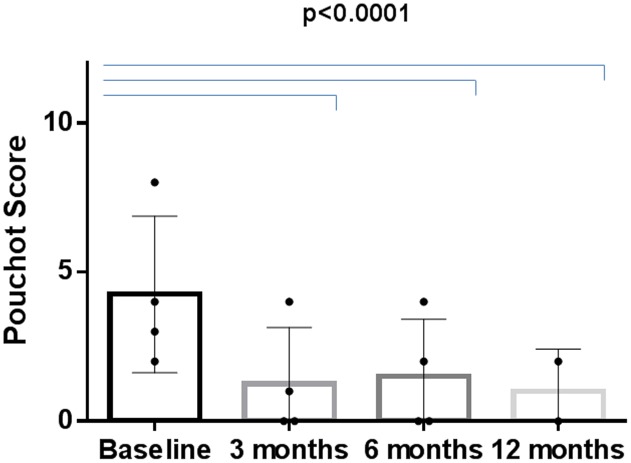
Modifications in Pouchot’s score changes during the 12-months study period in the CAN-treated patients.

After 3 months of CAN therapy, Pouchot’s score fell significantly from 4.25 ± 2.6 (range 2–8) to 1.25 ± 1.8 (range 1–4) (*p* < 0.0001) (**Figure 5**). Despite a general improvement in symptoms, therapy was discontinued in the chronic articular AOSD patient after 9 months because fever, arthritis and lymphadenopathy persisted over time. We were thus able to evaluate clinical parameters at 12 months in only three patients (**Table 8**). In one case, after 6 months of therapy, disease symptoms flared up and led to a MAS episode which was promptly treated and cured. The patient continued CAN therapy after that episode, and treatment is still ongoing. Signs of arthritis were observed at baseline in 2/4 patients; improvement was noted after 3 months in only one patient who experienced a complete remission during the follow-up. In all three remaining cases, there was no evidence at 12 months of clinical disease signs (no fever, rash, arthritis, or lymphadenopathy).

**Table 8:** Clinical and laboratory features at baseline and during the 12-months study period in the CAN-treated patients.

Clinical and laboratory	Baseline (*n* = 4) (%)	3 months (*n* = 4) (%)	6 months (*n* = 4) (%)	12 months (*n* = 3) (%)
Fever	100	25	50	0
Rash	50	0	0	0
Pleuritis	25	0	0	0
Pneumonitis	0	0	0	0
Pericarditis	25	0%	0	0
Lymphadenopathy	50	25	0	0
Hepatomegaly or increased liver enzymes	25	25	25	33.3
Hyperferritinemia	50	0	25%	33.3
Leucocytosis	100	25	25%	0
Sore throat	25	0	0%	0
Milagia	100	25	25%	0
Arthritis	50	25	50%	0
Macrophage Activation Syndrome (MAS)	0	0	25%	0


#### The Laboratory Efficacy of CAN

Serum ferritin levels were increased at baseline in 2/4 patients and were normalized after 3 months in all four. Serum ferritin levels were increased after 6 months in one patient who experienced a MAS episode. As therapy was discontinued in one patient, laboratory parameters after 12 months were assessed only in 3. Ferritin levels in 2 of these appeared normal; in the third (the same patient who experienced MAS 6 months earlier) ferritin rose to 425 ng/ml. That same patient presented altered liver enzyme, ESR, and CRP values throughout the observation period. The transaminase levels were normal during follow-up in the other three cases. ESR was elevated at baseline and at the end of the third month in 3 of the 4 patients; it was reduced in one patient after 6 months, and it was reduced in another after 12 months. CRP was also higher at baseline and after 3 months in all of the patients, it was decreased in two patients at the 6 months time point, and in another at the 12-months time point.

#### The Impact of CAN on Concomitant Therapies during Follow Up

Although no patient discontinued steroids during the 12 months study period, the mean PDN dosage was significantly lower with respect to the baseline value (143.75 ± 238.23 mg) as early as at 3 months (8.2 ± 7.8 mg, *p* < 0.0001) and at 12 months (10 ± 7.07 mg, *p* < 0.0001) (**Table 9**). The concomitant use of DMARDs was similar at baseline (2/4 cases) and at the end of the 12 months follow-up period (1/3 cases) (**Table 9**). Information regarding the current therapy and the last follow-up are outlined in **Table 7**.

**Table 9:** Changes in concomitant therapy strategies during the 12-months study period in the CAN-treated patients.

Therapy	Baseline (*n* = 4)	3 months (*n* = 4)	6 months (*n* = 4)	12 months (*n* = 3)
Steroids	100%	100%	100%	100%
	143.7 ± 238.2 mg	8.2 ± 7.8 mg	16.2 ± 13 mg	10 ± 7 mg
DMARDs	50%	50%	50%	33%


## Discussion

To our knowledge, this is the largest retrospective observational study evaluating the efficacy and safety of ANA and CAN in AOSD. Consistent with other studies, our data have confirmed the efficacy of IL-1-INH treatment in AOSD patients. It is well known that blocking IL-1, particularly IL-1β, represents standard therapy for a number of autoinflammatory conditions in which this cytokine plays a pivotal role. As far as IL-1α or IL-1β signal cascades are concerned, upon binding to the ligand-binding chain (IL-1RI), activation of the signaling pathway originates from the cytoplasmic Toll/IL-1 receptor (TIR) domain that associates with a TIR domain-containing adaptor, MyD88. Subsequent phosphorylation of several kinases leads to translocation of NF-κB to the nucleus and final expression of a large portfolio of inflammatory genes (Weber et al., 2010). The IL-1 family comprises 11 members (Dinarello, 2011), and some investigators (Colafrancesco et al., 2012; Priori et al., 2014) have shown that IL-1α/β and IL-18 are crucial in the pathogenesis of AOSD and are valid serological biomarkers of the disease (Colafrancesco et al., 2015).

First described as a treatment for AOSD in 2003, ANA, a recombinant version of the interleukin 1 receptor antagonist (IL1-RA), was the first IL-1 inhibitor used in clinical practice (Rudinskaya and Trock, 2003). Although the efficacy of ANA in AOSD has been described by several case-reports and case-series, due to the disease’s rarity, large randomized control trials (RCT) are still lacking. A meta-analysis published in 2014 identified eight studies, including one RCT (Nordström et al., 2012) demonstrating that ANA seems to be effective in improving AOSD manifestations and in reducing mean steroid dosage over time in patients refractory to conventional therapies (Hong et al., 2014). That meta-analysis, which considered all studies published between 2010 and 2014 (sample size ranging from 6 to 28 patients for a total of 134 patients receiving ANA treatment (100 mg/day), showed an overall and complete remission rate of 81.66 and 66.75%, respectively, thus supporting the efficacy of ANA in more than half of the patients studied.

The largest study until now evaluating ANA efficacy, which analyzed 41 patients, was published in 2015. Approximately 68% of the patients were found to be responders; half achieved complete remission defined as the total disappearance of signs and symptoms of disease and normalization of laboratory parameters. As reported in previous reports summarized in **Table 10** (Lequerré et al., 2008; Laskari et al., 2011; Nordström et al., 2012; Giampietro et al., 2013; Gerfaud-Valentin et al., 2014b; Cavalli et al., 2015; Ortiz-Sanjuán et al., 2015; Rossi-Semerano et al., 2015), a complete remission was demonstrated in up to 80% of the patients studied.

**Table 10:** Studies on AOSD patients treated with interleukin-1 (IL-1) inhibitors published in the past.

	Lequerré et al., 2008	Laskari et al., 2011	Nordström et al., 2012	Giampietro et al., 2013	Gerfaud-Valentin et al., 2014b	Cavalli et al., 2015	Ortiz-Sanjuán et al., 2015	Rossi-Semerano et al., 2015
N° patients	15	25	12	28	6	20	41	35
N° patients *Complete response (Remission^∗^)*	11 (73.3%)	20 (80%)	6 (50%)	15 (53.5%)	5 (83.3%)	14 (70%)	14 (34.1%)	16 (45.7%)
N° patients *Partial response^∗∗^*	2 (13.3%)	4 (16%)	N/A	4 (14.2%)	0	2 (10%)	14 (34.1%)	–
N° patients *Not effective^∗∗∗^*	2 (13.3%)	1 (4%)	1	6 (21.4%)	1 (16.6%)	4 (20%)	7 (17.07%)	14 (40%)
Discontinued for reasons other than remission/AE	–	–	–	1 (3.5%) (pregnancy)	–	–	1 (2.4%) (pregnancy)	2 (5.7%) (at patient’s request)
Steroid dosages reduced	Yes	Yes	Yes	Yes	Yes	Yes	Yes	N/A
AE	2 (13.3%) Cutaneous reactions	3 (12%) Cutaneous reactions	11 (91.6%) Cutaneous reactions	2 (7.1%) Cutaneous reactions	0	2 (10%) Cutaneous reactions	8 (19.5%) Cutaneous reactions	3 (8.5%) Infections (pneumonia, 1 VZV reactivation, MAS and infection)
		7 (28%) Infections (2 RTI, 3 UTI, 1 GE, 1 tissue abscess)				2 (10%) VZV Reactivation	4 (9.7%) Infections (1 osteomyelitis, 1 RTI, 2 UTI, 1 VZV reactivation) 1 (2.4%) Leukopenia 1 (2.4%) myopahy	


*The times therapy efficacy were evaluated were different in the various studies. ^∗^Remission: no fever (<37°C), no increase in inflammatory markers (ESR, CRP or ferritin), no tender or swollen joints. ^∗∗^Partial response: clinical improvement without complete symptom improvement or complete normalization of inflammatory markers. ^∗∗∗^Not effective: AOSD symptoms were unmodified during ANA treatment, including primary or secondary failure. RTI = respiratory tract infection; UTI, urinary tract infection; GE, gastroenteritis; VZV, varicella zoster virus; MAS, macrophage activation syndrome.*

In our cohort of 140 patients a prompt response to IL-1 inhibitors was demonstrated already after 3 months. Both clinical and laboratory parameters dramatically improved and remission was sustained over time and led to discontinuation of treatment in 28% of the cases during the follow-up. Most of the patients succeeded in achieving remission within 3 months of beginning therapy, a finding that is in agreement with reports in the literature describing the normalization of clinical, hematologic and biochemical parameters within hours to days after the first ANA injection. Pouchot’s score revealed a dramatic improvement in patients’ general clinical condition within 3 months time. Although the score lacks scientific validation, it is commonly used to evaluate patients’ disease activity. Some investigators have proposed using it as a “severity” rather than “activity” score. Others have suggested giving it a prognostic value and of using the score of 7.0 as a cut-off at the time of diagnosis as predictive of a more severe outcome and an increased risk of mortality (Ruscitti et al., 2016).

Interestingly, response to therapy was achieved rapidly in the same way in the systemic and chronic articular patients studied as far as the different signs and symptoms of the disease were concerned.

The number of articular patients with arthritis symptoms as well as the mean DAS 28 value significantly ameliorated over the time. Although this aspect seems to be confirmed by other studies (Laskari et al., 2011; Gerfaud-Valentin et al., 2014a), the efficacy of ANA in improving articular manifestations continues to be controversial. Laskari et al. (2011) demonstrated an improvement in joint manifestations (evaluated by ACR50 and ACR70 response) in 93 and 87% of their patients, respectively. Although a very low number of treated patients (4 with a systemic polyciclic pattern and 2 with a chronic articular one), no difference in response to therapy was identified in the patient groups by the study conducted by Gerfaud-Valentin et al. We also reported on the apparent efficacy of ANA in 3 patients with AOSD who was refractory to conventional therapies and who showed marked improvement in joint manifestations after therapy was begun (Priori et al., 2008). According to a study by Cavalli et al. (2015), ANA proved to be more effective in patients with the systemic disease pattern with respect to the chronic articular one, especially in those cases experiencing severe complications such as MAS.

The increased efficacy of ANA in patients experiencing a prevalently systemic involvement is in line with a dichotomous view of AOSD. Indeed, many assume that the systemic form is mainly driven by IL-1 responsible for fever or increased serum inflammatory marker, while the articular pattern is more similar to rheumatoid arthritis and may be principally driven by TNF-α (Maria et al., 2014). Likewise, in a study carried out by Giampietro et al. (2013) the most impressive response was obtained in the systemic AOSD pattern; a slightly less dramatic effect was noted in the chronic articular phenotype. Ortiz-Sanjuán et al. (2015) likewise reported a persistence of joint involvement after one year of treatment in 41.5% of the patients studied. Definitive conclusions on the real effectiveness of ANA on joint involvement have yet to be drawn.

We did not observe any differences in the type of response in the two patterns of the disease in our study nor when the patients were stratified according to age, sex, and previous types of therapies. Interestingly, no differences were noted in the AOSD group receiving monotherapy with respect to those who were also receiving DMARDs. The fact that similar results were reported by Giampietro et al. (2013) seems to confirm the relevance of taking into consideration the cost-effectiveness of ANA in view of its efficacy as monotherapy and its steroid sparing effect. For the time being, there is no consensus on this issue. Although the differences were not significant in a study by Ortiz-Sanjuán et al. (2015) focusing on the use of ANA in combination with conventional immunosuppressive drugs, it did not appear to improve the systemic symptoms or joint manifestations more efficaciously than did the monotherapy. ANA seems to spare steroids with the non-negligible consequence of preventing complications attributable to the chronic intake of steroids.

In most published studies ANA was used at the standard dose of 100 mg per day. In our study, a few of the patients began with lower dosages (50 mg per day or 100 mg every other day). Those patients were for the most part the ones who later needed dosage upgrade to the conventional one. We also reported on one patient whose 100 mg per day dosage did not appear to be sufficiently effective; when dosage was later switched to 200 mg/day the patient showed a good response and tolerability. In the presence of a clinical improvement, ANA dose has been reduced in several cases of our cohort, more commonly than in other reports (Laskari et al., 2011; Gerfaud-Valentin et al., 2014b; Ortiz-Sanjuán et al., 2015).

The optimal duration of ANA treatment in AOSD has not yet been established. As we recently reported in another multicenter study, the off-label use of IL-1 inhibitors presents a wide variability. While they are more frequently employed at a dosage based on body weight in pediatric patients, in adults a standard dose of 100 mg is frequently used (Vitale et al., 2016). Contrary to our observations, Kötter et al. described a recurrence in disease activity after reducing ANA administration to alternate days (Kötter et al., 2007). During a previous study focusing on the use of IL-1 inhibitors in different conditions, we noted that adjusting the dosage by increasing the dose at each administration or decreasing the timing between injections proved to be successful in 66.7% of patients. With one exception, we did not report any relapse after therapy was discontinued because of remission. In agreement with other reports, tapering of dosage was well tolerated and there was no evidence of relapse (Laskari et al., 2011; Giampietro et al., 2013; Ortiz-Sanjuán et al., 2015; Vitale et al., 2016).

The current study was able to retrospectively evaluate not only the efficacy but also the safety of ANA. In agreement with our previous reports (Sfriso et al., 2016; Vitale et al., 2016) and those of others (outlined in **Table 10**), ANA appears to be a safe drug that does not pose risks of infections. In the current study, the frequency of AEs was higher (33.5%) then that previously reported (**Table 10**). The most relevant AEs noted were reactions at the injection site (28/140 cases, 20%) or diffuse allergic skin rashes (12/140 cases, 8.6%); in some cases, the AEs were so severe as to determine therapy discontinuation. Although with a lower frequency, cutaneous reactions were the most commonly reported AE even in other reports (**Table 10**). In the light of the current study (the largest cohort of patients carried out until now) and the findings of others, the risk of reactions must be taken into consideration when therapy is being decided. The short half-life of ANA and the need for daily subcutaneous injections are other not negligible disadvantages that also need to be considered.

Canakinumab is a fully human monoclonal antibody against IL-1β with a longer half-life (26 days), meaning that it can be administered every 8 weeks. It has been approved for periodic fevers such as Cryopyrin Associated Periodic Syndromes (CAPS) as well as for SJIA. Promising results have recently been reported in patients with AOSD refractory to ANA. The first report, which was published in 2012 (Kontzias and Efthimiou, 2012), was followed by others (Barsotti et al., 2014; Lo Gullo et al., 2014). Although a study by Rossi-Semerano et al. (2015) did not report any significant differences in AEs in the ANA and CAN therapy groups, no AEs occurred in our 4 patients who were treated with CAN. Indeed, no cutaneous reactions or infections of any kind were noted. CAN proved to be effective in 3/4 patients and perhaps responsible for therapy discontinuation due to complete remission in one. It should be underlined that the failure took place in a patient with a chronic articular pattern. Although a slight improvement in the patient’s general condition was noted in that case, neither arthritis nor fever responded to therapy. In another case regarding a patient with a systemic pattern but also showing signs of arthritis, CAN proved to be efficacious. Definite conclusions concerning CAN efficacy in inflammatory joint involvement are not yet possible.

One patient also experienced MAS during therapy but completely recovered and subsequently continued therapy. Episodes of MAS also occurred during ANA treatment, in two cases with fatal outcomes. Although the percentage of patients experiencing recrudescence of the disease with severe life-threatening complications was quite low [6/140 (4.1%) = 5 cases during ANA treatment and 1 during CAN therapy], neither of the IL-1 inhibitors appeared to be capable of completely controlling these severe systemic manifestations.

Although the retrospective design and the open-label nature constitute potential limitations, the study represents the largest evaluation of IL-1 inhibition efficacy and safety in patients with AOSD. According to our findings, IL-1-INH seem to represent the best bDMARDs available and should be considered the first-line biologic treatment for patients not responding to conventional treatment. ANA can also be utilized as a monotherapy since it seems to have the same efficacy even when it is not associated to DMARDs. As ANA seems to be effective in improving all the clinical and laboratory features of AOSD, its use seems opportune regardless of the disease pattern. ANA also appears to be safe as far as the infectious risk is concerned. It should nevertheless be remember that development of skin reactions can at times be so invalidating as to induce therapy discontinuation.

Canakinumab also appears to be effective in AOSD, but in the current study it was possible to evaluate its efficacy only in patients who did not respond to ANA therapy or in whom ANA efficacy was lost. It cannot be excluded that those cases were more aggressive ones. It was also impossible to make any comparisons between ANA and CAN given the overwhelming differences in numbers.

## Conclusion

Interleukin-1 inhibitors appear to be a highly effective treatment of AOSD. Although most data regarding the effect of IL-1-INH in AOSD concern ANA, case reports focusing on treatment with CAN suggest that it has a similar efficacy. Further studies and randomized controlled trials are of course warranted.

## Author Contributions

SC, RP, PG, and PS drafted the manuscript. SC and RP carried out the data analysis. All the authors contributed to the enrollment process, in monitoring the patients, and in data collection. The study was conceived and designed by LC and PS.

## Conflict of Interest Statement

The authors declare that the research was conducted in the absence of any commercial or financial relationships that could be construed as a potential conflict of interest. The reviewer GP and handling Editor declared their shared affiliation, and the handling Editor states that the process nevertheless met the standards of a fair and objective review.
